# Immunomodulatory and anti-inflammatory therapeutic potential of gingerols and their nanoformulations

**DOI:** 10.3389/fphar.2022.902551

**Published:** 2022-09-05

**Authors:** Çiğdem Yücel, Gökçe Şeker Karatoprak, Özlem Bahadır Açıkara, Esra Küpeli Akkol, Timur Hakan Barak, Eduardo Sobarzo-Sánchez, Michael Aschner, Samira Shirooie

**Affiliations:** ^1^ Department of Pharmaceutical Technology, Faculty of Pharmacy, Erciyes University, Kayseri, Turkey; ^2^ Department of Pharmacognosy, Faculty of Pharmacy, Erciyes University, Kayseri, Turkey; ^3^ Department of Pharmacognosy, Faculty of Pharmacy, Ankara University, Ankara, Turkey; ^4^ Department of Pharmacognosy, Faculty of Pharmacy, Gazi University, Ankara, Turkey; ^5^ Department of Pharmacognosy, Faculty of Pharmacy, Acıbadem Mehmet Ali Aydınlar University, Istanbul, Turkey; ^6^ Department of Organic Chemistry, Faculty of Pharmacy, University of Santiago de Compostela, Santiago de Compostela, Spain; ^7^ Instituto de Investigación y Postgrado, Facultad de Ciencias de la Salud, Universidad Central de Chile, Santiago, Chile; ^8^ Department of Molecular Pharmacology, Albert Einstein College of Medicine, Park Avenue Bronx, NY, United States; ^9^ Pharmaceutical Sciences Research Center, Health Institute, Kermanshah University of Medical Sciences, Kermanshah, Iran

**Keywords:** cytokine, immunomodulatory, ginger, gingerol, *Zingiber officinale*

## Abstract

Ginger (*Zingiber officinale* Roscoe), a member of the Zingiberaceae family, is one of the most popular spices worldwide, known since ancient times, and used both as a spice and a medicinal plant. The phenolic compounds found in ginger are predominantly gingerols, shogaols, and paradols. Gingerols are the major phenolic compounds found in fresh ginger and contain mainly 6-gingerol as well as 4-, 5-, 8-, 10-, and 12-gingerols. Gingerols possess a wide array of bioactivities, such as antioxidant and anticancer, among others. Regarding the different array of biological activities and published data on the mechanisms underlying its action, the complex interaction between three key events, including inflammation, oxidative stress, and immunity, appears to contribute to a plethora of pharmacological activities of this compound. Among these, the immunomodulatory properties of these compounds, which attract attention due to their effects on the immune system, have been the focus of many studies. Gingerols can alleviate inflammation given their ability to inhibit the activation of protein kinase B (Akt) and nuclear factor kappa B (NF-κB) signaling pathways, causing a decrease in proinflammatory and an increase in anti-inflammatory cytokines. However, given their low bioavailability, it is necessary to develop new and more effective strategies for treatment with gingerols. In order to overcome this problem, recent studies have addressed new drug delivery systems containing gingerols. In this review, the immunomodulatory activities of gingerol and its underlying mechanisms of action combined with the contributions of developed nanodrug delivery systems to this activity will be examined.

## Introduction

Ginger, the dried rhizome of the plant *Zingiber officinale* Roscoe (Zingiberaceae), is a commonly used spice worldwide. It has been known as a folk medicine for thousands of years. Some regulatory authorities classify ginger as a safe herbal supplement (Vemuri et al., 2017), and it has been used both in complementary and alternative medicine preparations for the management of fevers, colds, and headaches, as an appetite stimulant, antibacterial, antiviral, antidiarrheal, choleretic, anti-emetic, and expectorant, to name a few (Dugasani et al., 2010; Embuscado, 2015; Konmun et al., 2017; de Menezes de Lima et al., 2018; Liang et al., 2018; Lashgari et al., 2022).

There are more than 200 identified compounds in ginger, and its bioactive constituents include tannins, anthocyanins, terpenes (e.g., α-zingiberene, β-bisabolene, β-sesquiphellandrene, ar-curcumene, or (E,E)-α-farnesene) and phenolic compounds (gingerols, paradols, shogaols, and zingerone) (Semwal et al., 2015; Koch et al., 2017; Mao et al., 2019). Gingerols are the major pungent compounds of ginger (Ozkur et al., 2022). Numerous studies have focused on ginger extracts and the phenolic compounds isolated from them, showing that gingerols have various pharmacological effects, mainly 6-gingerol. These include antiemetic, anti-inflammatory, antinociceptive, antioxidant, antimicrobial, anti-cancer, anti-hyperglycemic, anti-arteriosclerotic, rubefacient, digestive, and laxative effects (Semwal et al., 2015; Hitomi et al., 2017; Samad et al., 2017; Wang et al., 2018a; Ghasemzadeh et al., 2018; Han et al., 2018; Ho and Chang, 2018; Lee et al., 2018; Smith et al., 2018; Han et al., 2022a; Lashgari et al., 2022).

Among these effects, the immunomodulatory properties of these compounds have attracted attention owing to their effects on the immune system (Hitomi et al., 2017; Samad et al., 2017). Gingerols have been shown to inhibit protein kinase B (Akt) and nuclear factor kappa B (NF-κB) activation, thus causing an increase in anti-inflammatory cytokines and a decrease in proinflammatory cytokines (Guleria et al., 2022; Ley-Martínez et al., 2022).

There are few studies on the pharmacokinetics of 6-, 8-, and 10-gingerols and 6-shogaol (Lashgari et al., 2022). A small number of ginger compounds addressed their tissue distribution. Extracted or isolated compounds are evaluated in current studies by oral or intravenous administration in rodents and *in vitro* tests. The low absorption and rapid metabolism of 6-gingerol after oral administration are related to its poor solubility. To eliminate this problem, studies focusing on new drug carrier systems containing gingerol are important. Several studies evaluated the utility of different carrier solutions for efficient delivery of ginger compounds (Yu et al., 2011; Sato et al., 2017; Wang et al., 2018b; Ogino et al., 2018). *In vivo* studies investigating tissue distribution have shown that 6-gingerol is distributed to the lungs, brain, heart, liver, and kidneys, with the highest concentration in the gastrointestinal tract. After oral administration in rats, it has been determined that 6-gingerol is converted to glucuronide conjugates and its polar metabolites are excreted in small amounts in urine, and the compound is rapidly removed from the plasma after i.v. administration. Studies showing that 6-gingerol is generally excreted by the liver have determined that the elimination half-life of 6-gingerol is significantly increased in liver-damaged rats (Yu et al., 2011; Semwal et al., 2015; Dhanik et al., 2017; Mukkavilli et al., 2017; Arablou and Aryaeian, 2018). While 6-shogaol is rapidly absorbed and eliminated after oral administration, it is converted into glucuronide form in the blood, which significantly reduces its bioactivity (Kou et al., 2018). As gingerols have low bioavailability, it is necessary to develop effective and new approaches suitable for clinical use.

In the treatment of inflammatory and immune-related diseases resulting from defects or disorders of the immune system, modulation of the immune response is necessary. Immunomodulators are substances used to modulate components of the immune system, and there are many chemical immunomodulators on the market that treat inflammatory diseases. However, many of them have side effects. To search for safer drugs, natural-origin compounds come to the fore in the search for new drugs, and the traditional uses of these compounds also shed light on this research (Yuandani et al., 2021). The interest in gingerols, which are the subject of such studies, is increasing day by day; therefore, in this review, the immunomodulatory activities of gingerol and its underlying mechanisms along with the contributions of newly developed nanodrug delivery systems will be discussed.

## Overview on gingerols

**FIGURE 1:**
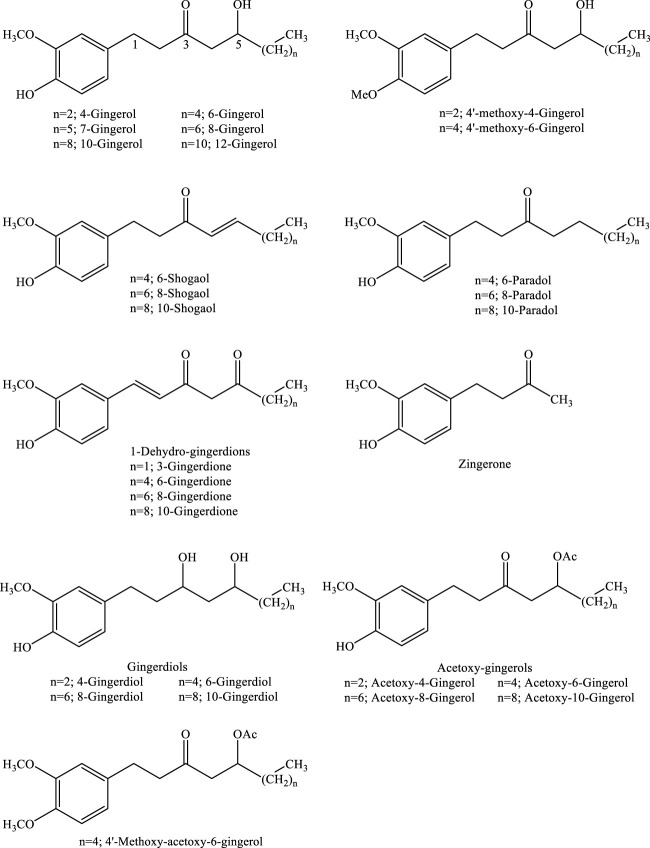
Chemical structures of gingerols and gingerol-related compounds.

Gingerols (23–25%), shogaols (18–25%), and related ketone derivatives are bioactive constituents of ginger (Grzanna et al., 2005). Gingerols are aromatic phenolic structures composed of a series of structural analogs of 1-(3-methoxy-4-hydroxyphenyl) 3-oxo-5-hydroxy-hexane, bearing different lengths of the unbranched alkyl side chain (Denniff et al., 1980; de Menezes de Lima et al., 2018; Li et al., 2019; Mao et al., 2019; Alolga et al., 2022). Derivatives of gingerols such as 4-, 6-, 7-, 8-, 10-, and 12-gingerols are differentiated based on their unbranched alkyl side chain length (Figure 1).

**FIGURE 2:**
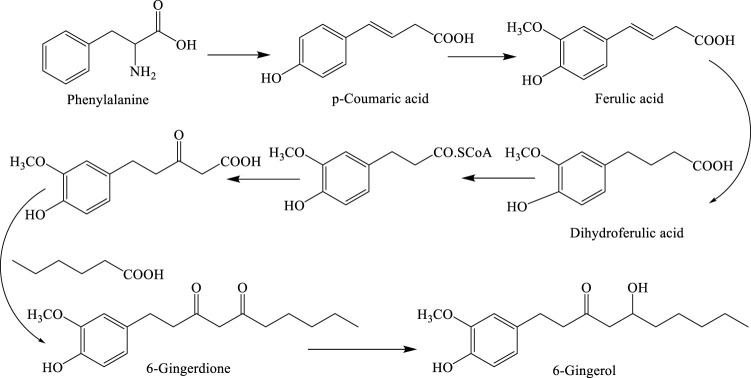
Biosynthetic pathway for 6-gingerol.

6-Gingerol ((5S))-5-hydroxy-1-(4-hydroxy-3-methoxyphenyl) decan-3-one), which was the first isolated compound from ginger rhizome, is the most abundant structure. This is the main component associated with the pungent taste of fresh ginger, while the other gingerols are present relatively in small quantities. However, gingerols are thermally unstable structures due to the presence of a β-unsaturated ketone group. Drying or heating of ginger rhizome results in the production of shogaol by dehydration of C-4 and C-5 positions. In spite of the overwhelming evidence on the conversion of gingerols to shogaols, once isolated, gingerols are stable in ethanolic solution over a 5-month period when stored at 4°C. Shogaols are formed easily from the corresponding gingerols, and their structures are shown in Figure 2 (de Menezes de Lima et al., 2018; Habtemariam, 2019; Li et al., 2019; Liu et al., 2019; Loung et al., 2019; Kukula-Koch and Czernicka, 2020; Unuofin et al., 2021).

Phenylalanine is converted to dihydroferulic acid, which subsequently participates in a biological Claisen reaction with malonate and hexanoate to form 6-dehydrogingerdione, which is finally converted to 6-gingerol. Shogaols, known as gingerol-related compounds, are responsible for the pungent and spicy–sweet taste of dried ginger. 6-Shogaol ((E)-1-(4-hydroxy-3-methoxyphenyl) dec-4-en-3-one), 8-shogaol ((E)-1-(4-hydroxy-3-methoxyphenyl) dodec-4-en-3-one), 10-shogaol ((E)-1-(4-hydroxy-3-methoxyphenyl) tetradec-4-en-3-one), and 12-shogaol are the main components in the rhizome. In nutraceutical preparations consisting of powdered or cooked ginger, the active compounds are shogaols. Which of the shogaols and/or gingerols plays the most important pharmacological role (Habtemariam, 2019; Zhang et al., 2021) has yet to be determined.

Paradols, which contain β-hydroxyl substitution, are the other structural analogues of gingerols produced as a result of shogaol biotransformation. They are stable products under high temperature or low pH. Although paradols are minor components of ginger, infrequently encountered in fresh samples, due to the better bioavailability of 6-paradol than 6-shogaol, it has been posited that they possess biological activity *in vivo* (Habtemariam, 2019; Kukula-Koch and Czernicka, 2020; Unuofin et al., 2021). Gingerdions, the dehydrogenation products of hydroxylated β-ketones of gingerols, are additional components inherent to ginger. Gingerdions, such as 3-, 6-, 8-, and 10-gingerdione, have been isolated from ginger as examples of this class. Gingerdiols, on the other hand, are 3-hydroxyl derivatives of gingerols and are present in ginger with the conformation of 4-, 6-, 8-, and 10-gingerdiols. Acetyl derivatives of gingerols contain acetoxy-4-, -6-, -8-, or -10-gingerol along with the 4′-methoxyl derivative of acetoxy-6-gingerol. Additional derivatives include diarylheptanoids and other minor components. Diarylheptanoids are characterized by a backbone structure of 1,7-diphenylheptanes, comprising compounds of macrocyclic or linear structures. In addition, the well-known gingerols and associated products, other compounds with the dione analogue of gingerols, such as 1-dehydro-10-gingerdione and gingerenone A were also found in ginger (Habtemariam, 2019; Kukula-Koch and Czernicka, 2020).

The biosynthetic pathway of gingerol was described by Denniff et al. (1981). As shown in Figure 2, phenylalanine is converted to dihydroferulic acid which participates in the biological reaction of Claisen, malonate, and hexanoate for the production of 6-dehydrogingerdione followed by 6-gingerol as a final compound (Semwal et al., 2015).

**FIGURE 3:**
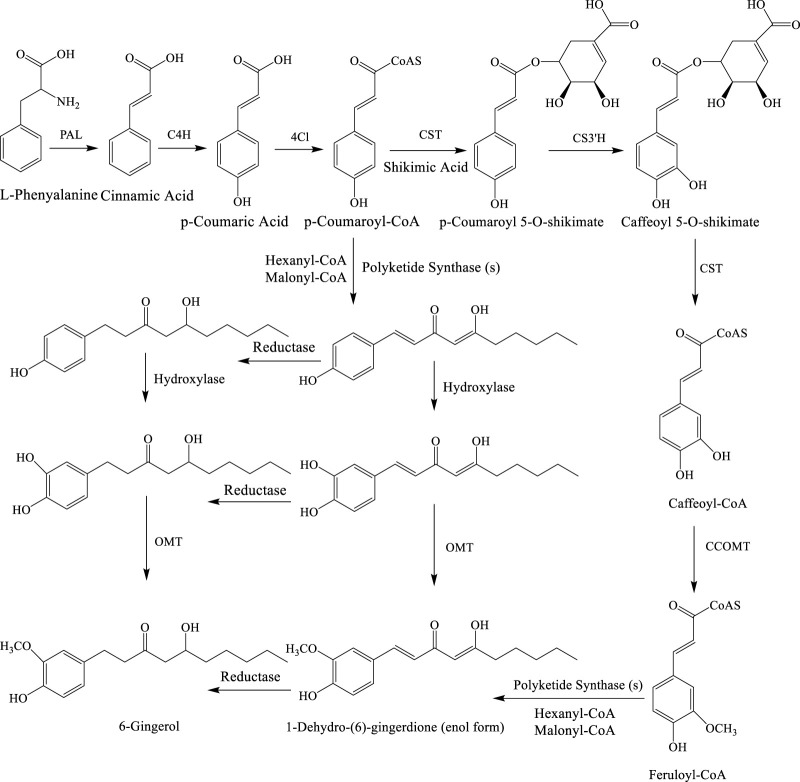
Alternative biosynthetic pathways for 6-gingerol. Enzymes: PAL, phenylalanine ammonialyase; C4H, cinnamate 4-hydroxylase; 4CL, 4-coumarate: CoA ligase; CST, p-coumaroyl shikimate transferase; CS3′H, p-coumaroyl 5-O-shikimate 3′-hydroxylase; OMT, O-methyltransferase; and CCOMT, caffeoyl-CoA O-methyltransferase. L-phenylalanine is converted into cinnamic acid *via* PAL. Next, it is turned into p-coumaric acid with the use of C4H. 4CL is then used to get p-coumaroyl-CoA. CST is the enzyme that is responsible for the binding of shikimic acid and p-coumaroyl-CoA. The complex is then selectively oxidized at C3 by CS3′H to alcohol. Upon further reaction with CST, shikimate dissociates from this intermediate, thereby yielding caffeoyl-CoA. To achieve the desired substitution pattern on the aromatic ring, CCOMT converts the hydroxyl group at C3 into methoxy as seen in feruloyl-CoA. 1-Dehydro-(6)-gingerdione and then 6-gingerol are formed from feruloyl-CoA by the enzyme polyketide synthase.

An alternative biosynthetic pathway was also suggested by Ramirez-Ahumada et al. (2006) with different enzymes including caffeic acid O-methyltransferase, caffeoyl-CoA-O-methyltransferase, p-coumaroyl shikimate transferase, phenylalanine ammonia lyase, and p-coumaryl quinate transferase (Figure 3) (Semwal et al., 2015).

## Interaction of gingerols with the immune system

The function and activity of the immune system, which maintains homeostasis in the healthy organism, is affected by various exogenous and endogenous agents, resulting in either immunosuppression or immunostimulation (Jantan et al., 2015). Many natural compounds affect the functions of immune cells or affect the secretion of antibodies to control infection and maintain immune balance (Ortuño-Sahagún et al., 2017). These compounds have been shown to activate innate immune components such as stimulation of macrophages and lymphocytes, modulate the cytokine profile, decrease the incidence of infection, and stimulate the apoptosis process (Nagoba and Davane, 2018).

The anti-inflammatory properties of ginger, which has been used in Ayurvedic medicine for its various pharmacological properties for a long time, have been known for centuries. In addition, a large portion of traditional Chinese herbal remedies contains ginger. Evidence of the suppressive effects of ginger on prostaglandin biosynthesis dating to the early 1970s has been repeatedly confirmed. These results showed that the natural-origin compounds contained in ginger exhibit similar pharmacological efficacy to non-steroidal anti-inflammatory drugs (Kumar et al., 2013; Aziz et al., 2015; Deng M. et al., 2022). It has also been explained that phosphatidylinositol-3-kinase (PI3K), Akt, and NF-κB light chain enhancer of activated B cells are prominent in the mechanism of the anti-inflammatory effect (Mao et al., 2019; Zahoor et al., 2020; Zhang et al., 2021; Zhou et al., 2022).

### *In vitro* studies

Gingerols have a versatile pharmacological profile comprising immune functions. In many studies, the immunomodulatory activities of gingerols, especially 6-gingerol, have been elucidated. In a study investigating the *in vitro* anti-hydatic and immunomodulatory effects of ginger and the major phenolic compound 6-gingerol in cystic echinococcosis, protoscoleces (PSC) and cyst wall were used. The activity of ginger extract and the major phenolic compound in conjunction with interferon-gamma (IFN-γ) on PSCS cultured with mononuclear cells of hydatic patients was examined, and nitric oxide (NO) generation was measured in both cultures. Concentration- and time-dependent cytotoxic effects on PSC and cyst wall were observed in ginger extract, while the activity of 6-gingerol was found to be lower. It has also been reported that the efficacy of 6-gingerol in reducing high NO affords protection against host cell death (Amri and Touil Boukoffa, 2016), as corroborated in other studies. This phenolic compound inhibited inducible NO synthase (iNOS) and tumor necrosis factor-α (TNF-α) expression by blocking NF-kB and protein kinase C (PKC) signaling pathways in mouse macrophages induced by LPS (lipopolysaccharide) (Lee et al., 2009). Ippoushi et al. showed that 6-gingerol (0.2–40 µM) at increased concentrations potently inhibited NO formation and significantly reduced iNOS in LPS-stimulated mouse macrophages (J774.1). Similarly, Tripathi et al. (2007) showed that 6-gingerol decreased the generation of iNOS, cyclooxygenase-2 (COX-2), and proinflammatory cytokines IL-1β, IL-12, and TNF-α in LPS-induced murine peritoneal macrophages. However, 6-gingerol did not affect the expression of B proteins 7.1 and 7.2 and histocompatibility complex II (MHC II). U937 cells were differentiated and subjected to LPS with or without 6-, 8-, 10-gingerols, and 6-shogaol for 24 h. The most pronounced activity in inhibiting prostaglandin E2 (PGE2) production and COX-2 activity was found for 10-gingerol, while 8- and 6-gingerol had lower activity. The inhibitory IC50 value of 6-shogaol on PGE2 production was found to be much higher than gingerols, and it was reported to be ineffective in blocking COX-2 expression (Lantz et al., 2007).

Tjendraputra et al. (2001) have shown the structure and anti-inflammatory activity relationship for gingerol derivatives. 6-, 8-, 10-, and 12-Gingerols, 8-gingerdiol, shogaols, and paradols were tested for their inhibitory activities of COX-2. 10-Gingerol, among the tested compounds including 6-, 8-, 10-, and 12-gingerols, displayed the highest inhibitory activity on COX-2, while the other gingerol derivatives, which have shorter and longer unbranched alkyl side chains, displayed moderately weaker inhibitory effects. The 14-carbon-length lipophilic alkyl side chain exhibited optimal inhibition of COX-2. Natural gingerols and their derivatives have the 4-hydroxyl and 3-methoxyl (1,3,4 trisubstituted aromatic ring system) as the general structure, which is essential for their biological activities. Differences in their potency have been attributed to their alkyl side chains. The lipophilicity of the alkyl side chain and the substitution of carbonyl and hydroxy groups of the side chains as well as methoxy and hydroxy groups on the aromatic moiety have been posited to be the structural determinants of COX-2 inhibitory efficacy of gingerol derivatives. The inhibitory effect of hydroxy and carbonyl group substitution on the alkyl side chain of COX-2 enzyme has yet to be determined. The presence of a carbonyl group at position C3 and an OH substitution at position C2 on the side chain increased the efficacy of the compound, when compared to OH substitution at position C5. Replacement of the carbonyl group at position C3 with a hydroxy group failed to change its efficacy. The presence of a hydroxy group at C5 enhanced its inhibitory activity, suggesting interaction with the enzyme through the formation of H-bonding and hydrophobic interactions. Regarding the aromatic moiety, the hydroxy substitution at C3 or C4 on the aromatic ring is essential for the inhibitory activity of COX-2. Additionally, a compound with no free phenolic hydroxy group displayed no significant inhibitory effect, suggesting that a free phenolic hydroxy group is essential for activity, probably through H-bonding to the binding site of the enzyme (Tjendraputra et al., 2001; Habtemariam, 2019).

Osteoblast-like MG63 cells were treated with TNF-α to create inflammatory conditions and treated with 6-gingerol. Osteogenic markers and cytokines were evaluated by enzyme-linked immunosorbent assay (ELISA), real-time polymerase chain reaction (PCR), and western blotting methods. An increased amount of IL-6 and increased NF-κB expression were noted in MG-63 cells induced with TNF-α, as a result of treatment with 1–50 µM concentrated 6-gingerol, and the rate of inflammation was reported to decrease. It was suggested that 6-gingerol might be a promising compound for the treatment of osteoporosis or bone inflammation given its effects on IL-6 in bone (Tripathi et al., 2007; Fan et al., 2015). The effects of 6-gingerol on the cellular and molecular mechanisms underlying osteoclast differentiation-associated inflammation have been elucidated. The mechanism appears to be associated with the downregulation of the receptor activator of NF-κB ligand (RANKL) expression in osteoblasts by inhibiting PGE2, thus inhibiting IL-1-stimulated osteoclast differentiation (Hwang et al., 2018). Accordingly, control of RANKL expression and downregulation of RANKL availability and its receptor osteoprotegerin may accomplish inflammation in osteoclast differentiation and slow bone decrement. The fact that 6-gingerol has these effects affords an advantage in treatment (Suda et al., 2004; Ha et al., 2006).

In a study on allergic rhinitis by Kawamoto et al. (2016), 6-gingerol inhibits the phosphorylation of mitogen-activated protein (MAP) kinases, calcium release, and nuclear localization of c-fos and NF-κB by phorbol myristate acetate (PMA) and ionomycin excitation. The authors posited that 6-gingerol acted by suppressing the differentiation of both Th1 cells and Th2 cells into naïve T cells as well as the production of cytokines for T-cell activation and proliferation, thereby preventing or alleviating the symptoms of allergic rhinitis without causing B-cell and mast cell activation.

Oleoresin, gingerol, and shogaol, the bioactive compounds of ginger, were evaluated in human lymphocytes. For natural killer (NK) cell analyses, cells were treated with paraquat and the compounds were applied at 50, 100, and 200 μg/ml concentrations. At 50 μg/ml concentration, oleoresin and gingerol induced B- and T-cell proliferation, with shogaol showing similar activity at high concentrations. In addition, NK-cell lysing activity in the presence of paraquat has been shown to be significantly increased by both oleoresin and gingerol (Zakaria-Rungkat et al., 2003). Dugasani et al. (2010)demonstrated that 6-shogaol was more potent in its anti-inflammatory activity compared to 6-gingerol, 8-gingerol, and 10-gingerol. At 6 µM concentration, 6-shogaol inhibited nitrite and PGE2 release by 80% and 87%, respectively. Among the gingerols, 10-gingerol was found to be more potent than 6- and 8-gingerols because of its carbon chain length.

T lymphocytes are relevant in the pathogenesis of some autoimmune diseases and other chronic inflammatory ailments. Bernard et al. (2015) focused on the effector functions of 6-gingerol, 8-gingerol, and 10-gingerol on T lymphocytes. Cytokine production and signaling *via* the IL-2 receptor were also evaluated. While all three gingerols inhibit DNA and interferon-γ synthesis of T lymphocytes, only 8-gingerol and 10-gingerol inhibited CD25 and CD69 expression and IL-2 synthesis. In the presence of 8- or 10-gingerol, exogenous IL-2 did not increase T-lymphocyte proliferation, but it was increased in the presence of 6-gingerol. CD25 expression was unaffected with 8-gingerol and 10-gingerol, but IL-2-stimulated proliferation of CTLL-2 cells was impaired. Both 10-gingerol and 8-gingerol inhibited T lymphocytes stronger than 6-gingerol.

The interaction of immune effector cells, local fibroblasts, and tissue macrophages determines liver damage and establishes a connection between chronic inflammation and fibrosis (Xu et al., 2012). 6-Gingerol has shown beneficial effects on liver fibrosis in rats. This effect is mediated by its antioxidant activity *via* reduced glutathione (GSH) depletion and lipid peroxide accumulation, as well as decreased expression of NF-κB, TNF-α, intercellular adhesion molecule (ICAM), toll-like receptor (TLR4), and vascular cell adhesion molecule (VCAM) (Algandaby et al., 2016). In an *in vitro* hepatic inflammatory model, HuH7 cells were induced with IL1β and treated with S-6-gingerol. S-6-gingerol decreased the mRNA levels of IL6, IL8, and serum amyloid A1 (SAA1), suppressed reactive oxygen species (ROS) formation, and decreased inflammation and oxidative stress by increasing mRNA levels of 24-dehydrocholesterol reductase (DHCR24). Furthermore, this compound reduced NF-κB activity as well as IL1β-induced upregulation of COX2 (Li et al., 2013).

Immunomodulators exhibit anticancer activity due to their anti-inflammatory, antioxidant, apoptotic, anti-angiogenesis, and anti-metastatic effects (Mohamed et al., 2017). Studies have shown that 6-gingerol targets molecular signaling pathways that could potentially be used to treat cancer. Bode et al. (2001) demonstrated that 6-gingerol inhibits the epidermal growth factor-induced activated activator protein 1 (AP-1), which has an important effect on tumor promotion. Cell migration and motility were significantly reduced at increasing concentrations in breast cancer (MDA-MB-231) cells treated with 6-gingerol. It was stated that the activities of MMP-2 (matrix metalloproteinase-2) and MMP-9 (matrix metalloproteinase-9) were inhibited after administration of this compound, and there was no change in MMP-9 protein amounts, while MMP-2 protein levels decreased. The effects of 6-gingerol on MMPs, which are possible mediators of invasion and metastasis in some cancer cells, have shown its ability to suppress adhesion, invasion, and motility in MDA-MB-231 cells (Lee et al., 2008). Xue et al. (2021)confirmed that 6-gingerol suppresses levels of MMP-2 by reducing nuclear levels of yes-associated protein (YAP), which acts as a promoter in tumor metastasis in many cancers. In another study, it was shown that 6-gingerol reduces the viability of human promyelocytic leukemia (HL-60) cells, has an inhibitory effect on DNA synthesis, and is responsible for apoptotic cell death (Lee and Surh, 1998). Wang et al. (2003) suggested that 6-gingerol causes DNA fragmentation in HL-60 cells and inhibits B-cell lymphoma 2 (Bcl-2) expression; thus, inhibition of Bcl-2 expression may be responsible for the mechanism of 6-gingerol-induced apoptosis. In another study, 6-gingerol combined with tumor necrosis factor-related apoptosis-inducing ligand (TRAIL) increased TRAIL-induced cell death in human lung cancer (A549) cells by an enhanced accumulation of microtubule-associated protein light chain 3-II and p62, establishing inhibition of autophagy flux (Nazim et al., 2015). In the study by Deng et al. (2022), it was also found that 6-gingerol increased cytotoxicity of CD8+T cells against tumor cells by promoting mitochondrial biogenesis in CTLL-2 (cytotoxic T cells) cells.

Han et al. (2022a) examined the antioxidant effect of 6-gingerol on H9c2 cells chemically induced by CoCl_2_ to mimic hypoxia-related cellular damage and its possible role in activating the endogenous major antioxidant, nuclear factor erythroid 2-related factor 2 (Nrf2) pathway, and p38/NF-κB signaling pathways. Their findings showed that the cytoprotective effect of 6-gingerol could potentially be related to the regulation of oxidative stress and apoptosis by activating the Nrf2 pathway and inhibiting the p38/NF-KB pathways (Han et al., 2022a). The same group has recently shown that 10-gingerol can protect against H9c2 cardiomyocyte damage induced by hypoxia/reoxygenation in *in vitro* experiments. They further investigated the protective effects of 10-gingerol in mice, assuming that it confers protective effects against myocardial ischemia damage and the potential mechanisms of action by examining histopathological lesions, analyzing expression levels of cardiac enzymes, *in vivo* oxidative stress and apoptosis, and proteins involved in the Janus kinase 2 (JAK2)/signal transducer and activator of the transcription 3 (STAT3) signaling pathway. Their results demonstrated the applicability of 10-gingerol to the treatment of cardiovascular disease (Han et al., 2022b).

Since chronic inflammation can trigger carcinogenesis, colorectal cancer (CRC) is promoted by tumor- and therapy-induced inflammation. T helper 1 (TH1)-cell-mediated immune responses and IFN levels in CRC tumors have been linked to a better prognosis, whereas TH17-cell-mediated immune responses have been linked to a worse prognosis, highlighting the importance of the T-cell response in limiting or driving tumor cell growth (Schmitt and Gretten, 2021). Studies have shown the potential of gingerols to be an effective chemopreventive agent for colorectal carcinoma with their anti-inflammatory properties. Lee et al. (2008) revealed that 6-gingerol generated substantial G2/M, G1 cell cycle arrest, and death in colorectal cancer cell lines LoVo and HCT116 by inhibiting cyclin D1, cyclin A, and cyclin B1. In HCT116 and HT29 colon cancer cells treated with ginger extract, it was observed a dose-dependent increase in apoptosis with a cell cycle arrest in G0/G1 and G2/M phases and a concomitant decrease in S-phase DNA synthesis (Abdullah et al., 2010). The fresh ginger extract containing gingerols caused cell death in HT29 colon cancer cells, which was related to the overexpression of caspase 9, a pro-apoptosis gene, and downregulation of Bcl-xL, an anti-apoptotic gene. The downregulation of cancer pathway genes such as Kirsten rat sarcoma virus (KRAS), extracellular signal-regulated kinase (ERK), AKT, and NF-κB (p65) was shown to be linked with the induction of the inhibitory gene IκBα (Tahir et al., 2015). The mechanism of apoptosis in SW-480 (colon cancer) cells of 6-gingerol is the phosphorylation of downregulated PMA, ERK1/2, and c-Jun N-terminal kinase (JNK), mitogen-activated protein kinases (MAPKs), and induction of the activation of the AP-1 transcription factor (Radhakrishnan et al., 2014). By targeting the epidermal growth factor receptor (EGFR)/signal transducer and activator of transcription/extracellular signal-regulated kinase pathway, 8-gingerol decreased CRC (HCT116 and DLD1cell lines) cell proliferation and migration, and the effects of 8-gingerol were dependent on EGFR expression (Hu et al., 2020). Because of the importance of leukotrienes in human cancer and chronic inflammation, as well as the overexpression of leukotriene A4 hydrolase in colorectal cancer, a study examined the effects of 6-gingerol. According to the findings, 6-gingerol inhibits anchorage-independent cancer cell proliferation in HCT116 colorectal cancer cells by reducing leukotriene A4 hydrolase (LTA4H) activity (Jeong et al., 2009).

**FIGURE 4:**
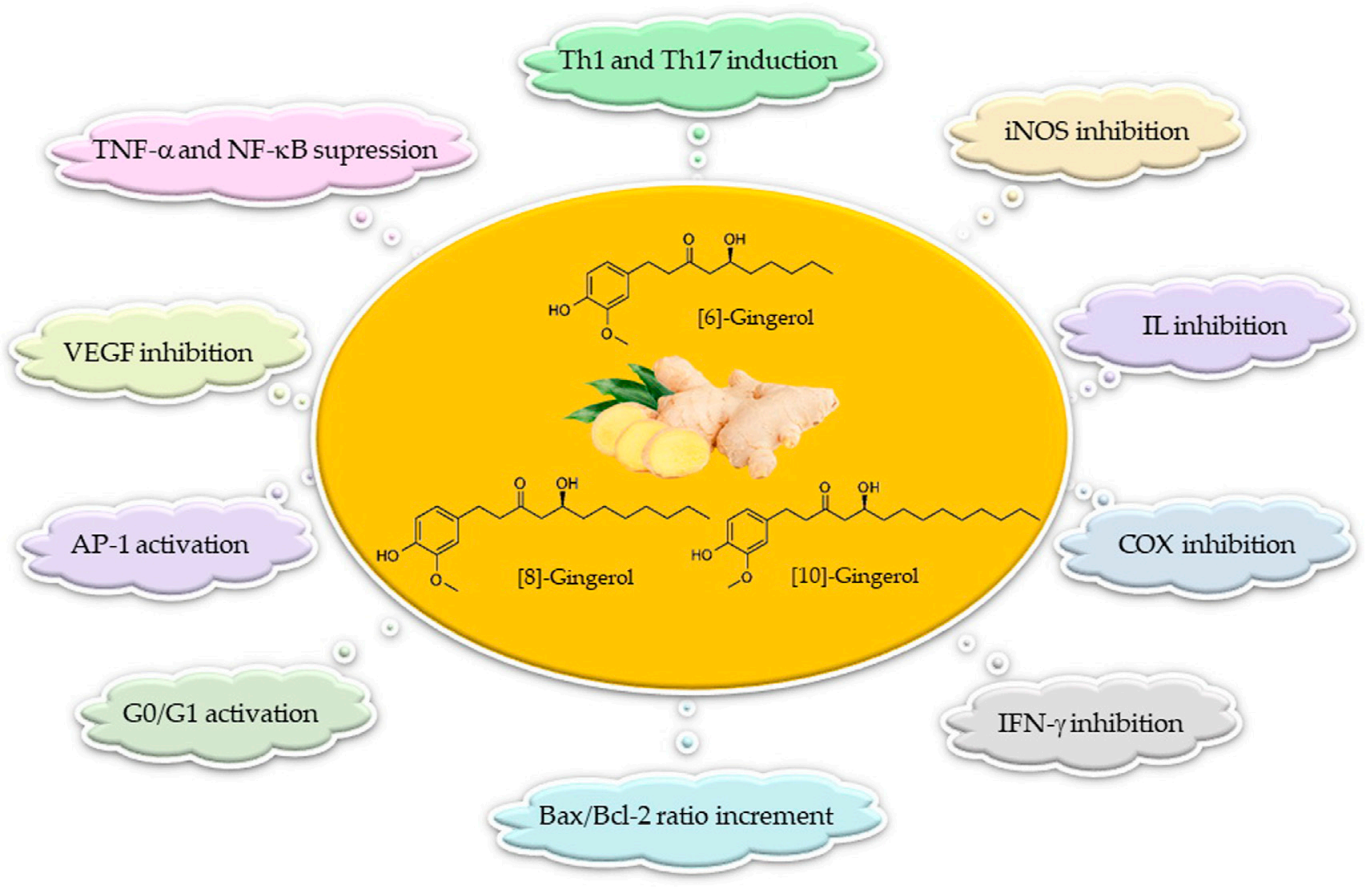
Molecular mechanisms in the immunomodulating activity of gingerols.

10-Gingerol has been proven cytotoxic in many tumor cells, such as A549, HCT15, SK-MEL-2, and SK-OV-3. The results show that at 50 μM or above concentrations, 10-gingerol leads to concentration-dependent cell death, but no cytotoxic effect was observed at lower concentrations (Kim et al., 2008). The effects of 10-gingerol on human colorectal cancer cells (SW480) were identified by Chen et al. (2009). It was reported that 10-gingerol induces [Ca^2+^] increment by evoking protein kinase C-independent Ca^2+^ release from the endoplasmic reticulum and Ca^2+^ influx from non-L-type Ca^2+^ channels. In a different study, 10-gingerol was assessed in SKOV-3, OVCAR3, and HEY ovarian cancer lines. The time- and dose-dependent decrease in the cell number of ovarian cancer cell lines treated with 10-gingerol was affected by the decrement in cell proliferation rather than a cytotoxic effect. The decreased proliferation was explained by an enhanced percentage of cells in the G2 phase of the cell cycle and a concerning decrease in the percentage of cells in G1. In addition, treatment of ovarian cancer cells with 10-gingerol caused decreased expression of cyclin A, B1, and D3 (Rasmussen et al., 2019). 10-Gingerol presented an increased Bax/Bcl-2 ratio in the human colon cancer (HCT116) cell line, which resulted in the activation of poly-ADP-ribose polymerase, caspase-3, and caspase-9, which are distinct features of apoptosis. In addition, other pathways accompanying apoptosis include phosphorylation of the MAPKS family, JNK, p38 MAPK (p38), and ERK (Ryu and Chung, 2015). The molecular mechanism of immunomodulation of gingerols is summarized in Figure 4. *In vitro* studies are summarized in Table 1.

**TABLE 1:** *In vitro* research reports of gingerols.

Compound	Subject	Concentration	Potential mechanism	Research (author and year)
6-Gingerol	Protoscoleces, cyst wall	100 μg/ml	• Reduced NO levels	Amri and Touil Boukoffa (2016)
			• Reduced cell viability	
6-Gingerol	RAW 264.7 macrophage cell line	20, 40, and 80 µM	• Inhibited iNOS and TNF-α expression	Lee et al. (2009)
			• Blocked NF-kB and PKC signaling pathways	
			• Prevented the intracellular Ca^2+^ overload induced by LPS	
			• Decreased MMP	
6-Gingerol	J774.1 macrophage cell line	0.2–40 µM	• Inhibited NO formation	Ippoushi et al. (2003)
			• Reduced iNOS	
6-Gingerol	Peritoneal macrophage	1, 10, and 100 ng/ml 1, 10, and 100 μg/ml	• Inhibition in NF-kB induction	Tripathi et al. (2007)
			• Decreased TNF-α, IL-12, and IL-1β	
			• No effect on costimulatory molecules (B7.1 and B7.2) and MHC II expression	
6-Gingerol, 8-gingerol, 10-gingerol	U937 pro-monocytic, human histiocytic lymphoma cell line	0.2–1 μg/ml	• Inhibition of PGE2 production (10-gingerol <8-gingerol<6-gingerol)	Lantz et al. (2007)
			• No effect on TNF-*α* production	
6-Gingerol, 8-gingerol, 10-gingerol, 12-gingerol	A549 adenocarcinoma human alveolar basal epithelial cells	1, 10, and 100 µM	• Highest inhibitory activity on COX-2 with 10-gingerol	Tjendraputra et al. (2001)
6-Gingerol	Osteoblast-like MG63 cells	1–50 µM	• Increased enzyme activity of ALP	Fan et al. (2015)
			• Increased collagen type I synthesis	
			• Decreased IL-6 levels	
			• Inhibition of NF-κB p65 nuclear translocation	
6-Gingerol	Cocultures of osteoblasts and osteoclast precursor cells	1–10 µM	• Inhibition of RANKL expression	Hwang et al. (2018)
			• Decreased PGE_2_ levels	
6-Gingerol	Jurkat (human T-cell line), Raji (human B-cell line)	6.25–50 µM	• Inhibited differentiation of both Th1 cells and Th2 cells	Kawamoto et al. (2016)
			• Suppressed phosphorylation of MAP kinases, calcium release, and nuclear localization of c-fos and NF-κB	
			• Decreased IL-2 levels	
6-Gingerol	Human lymphocytes obtained from a healthy male	50, 100, and 200 μg/ml	• Induced B- and T-cell proliferation	Zakaria-Rungkat et al. (2003)
			• Increased NK-cell lysing activity	
6-Gingerol, 8-gingerol, 10-gingerol	Raw 264.7 mouse macrophage cell line	6 µM	• Inhibition of nitrite release	Dugasani et al. (2010)
			• Inhibition ofPGE2 release	
			• 10-Gingerol was found to be more potent among the gingerols	
6-Gingerol, 8-gingerol, 10-gingerol	CTLL-2 CD8+ T lymphocyte	2.5–100 µM	• 8-Gingerol and 10-gingerol inhibited CD25 and CD69 expression and IL-2 synthesis	Bernard et al. (2015)
			• 10-Gingerol and 8-gingerol inhibited T lymphocytes stronger than 6-gingerol	
S-6-gingerol	HuH7 hepatoma cell line	100 µM	• Decreased IL6, IL8, and SAA1 levels	Li et al. (2013)
			• Suppressed ROS formation	
			• Reduced NF-κB activity and IL1β-induced upregulation of COX2	

### *In vivo* studies

In the research of Bhaskar et al. (2020), mice were infected with MDR, Mtb, H37Rv, and XDR strains to evaluate the effect of 6-gingerol in the tuberculosis model. In mice infected with *Mycobacterium tuberculosis*, 6-Gingerol inhibited mycobacterial growth in the lung, liver, and spleen. In addition, the results of cytokine measurements in peritoneal macrophages obtained from mice suggested the modulation of pro- and anti-inflammatory innate cytokines after 6-gingerol treatment. The number of CD4± and CD8+ T cells or Cd11b± and CD11c± cells induced with 6-gingerol treatment led to p38 MAPK phosphorylation. In addition, 6-gingerol has been reported to induce host-protective Th1 (type 1 T helper) and Th17 (T-helper 17) immune responses versus *Mycobacterium tuberculosis* infection. In a different study, 6-gingerol was discovered to effectively reduce LPS-induced inflammation in the mouse ileum by altering the NF-κB pathway. By reducing the expression of the genes for Bax and cytochrome c as well as by blocking the caspase-3 pathway, it also reduced apoptosis in the ileum (Guo et al., 2022).

In a rat model of sepsis, the activities of 6-gingerol and 10-gingerol were evaluated in the context of modulation of acute kidney injury and metabolic disruption. 6-Gingerol and 10-gingerol were injected (i.p) to rats at 25 mg/kg concentration. Sepsis enhanced the mRNA expression of inflammatory IL-1β and TNF-α markers, as well as transforming growth factor 1 (TGF-1), a biomarker of immune dysfunction. Treatment with gingerols led to a decrease in the transcription of these biomarkers. Both 6- and 10-gingerols restored kidney function by reducing oxidative/nitrosative stress and through antioxidant activity associated with proinflammatory cytokines. Gingerols decreased septic acute kidney injury by reducing kidney ailments, oxidative stress, and inflammatory response, possibly by a mechanism associated with enhanced dimethylamine and methylsulfonylmethane production (Rodrigues et al., 2018). These results are also supported by previous studies establishing that a gingerol-enriched fraction consisting of 6-gingerol and 10-gingerol attenuated the mRNA expression of IL-2, IL-β, TNF-α, IFN-γ proinflammatory cytokines, and ROS activation in an aminoglycoside-induced nephrotoxicity model (Rodrigues et al., 2014). In a different study, 6-gingerol was also shown to be able to decrease NO and immune cells population in house dust mite-treated mice (Ajayi et al., 2022).

8-Gingerol’s role as an immunosuppressant has been studied by Lu et al. (2011). 8-Gingerol suppressed splenocyte proliferation induced by LPS and concanavalin A (ConA) *in vitro*. It also showed immunosuppressive potency on the immune response to ovalbumin (OVA) in mice by decreasing the percentage of CD19^+^ B cells and CD3+T cells at 50 and 100 mg/kg doses and IgG, IgG1, and IgG2b levels were also decreased with the same doses. In a different study, a 400 mg/kg/day oral dose of 6-gingerol for 1 week showed immunomodulatory activity in mice sustained to 5 Gy c-radiation. In the treatment group, spleen relative weight and macrophage survival were increased, and splenocyte survival and proliferation decreased (Zhu, 2009).

6-Gingerol has demonstrated its immunomodulatory activity by acting on humoral and cell-mediated immune responses in rats. It was observed that oral application of 800 mg/kg bw 6-gingerol (7 days) increased the circulating antibody titer (88.2) and delayed-type hypersensitivity (3.5) compared to the control (8.9 and 0.2, respectively) group. In addition, it enhanced the humoral antibody response and increased cellular immunity by expediting the footpad thickness response to sheep red blood cells (RBCs) in rats immunized with sheep RBCs. As a result, this study demonstrated the capacity of 6-gingerol to increase lymphocyte proliferation (Farhath et al., 2013).

Ulcerative colitis is one of the most prevalent inflammatory bowel diseases, and three different types of drugs are presently used in clinical therapy: aminosalicylates, corticosteroids, and immunosuppressive agents. The efficacy of 6-gingerol, 8-gingerol, and 10-gingerol against ulcerative colitis in a rat model was investigated by Zhang et al. (2017). Dextran sulfate sodium (DSS) was preferred to induce colitis, and the compounds were administered i.p at doses of 30 mg/kg/day for 7 days. All three gingerols showed relatively equal activity in alleviating colitis symptoms, increasing superoxide dismutase activity, decreasing malondialdehyde levels in colon tissue and myeloperoxidase activity, and decreasing the amount of IL-1β and TNF-α in serum. Histological evaluations are consistent with the propensity of the three gingerols to accelerate mucosal injury healing. The effects of 6-gingerol on ulcerative colitis have also been addressed. 6-Gingerol inhibited the decrease of mRNA levels and serum and bowel amounts of IL-10, increased Th17 cells, and declined Treg cells stimulated by DSS and demonstrated analogous effects with the standard drug mesalazine (Sheng et al., 2020). Treatment with 6-gingerol reversed DSS-mediated weight reduction, diarrhea, rectal bleeding, and colonic contraction in ulcerative colitis BALB/c mice (Ajayi et al., 2015).

**FIGURE 5:**
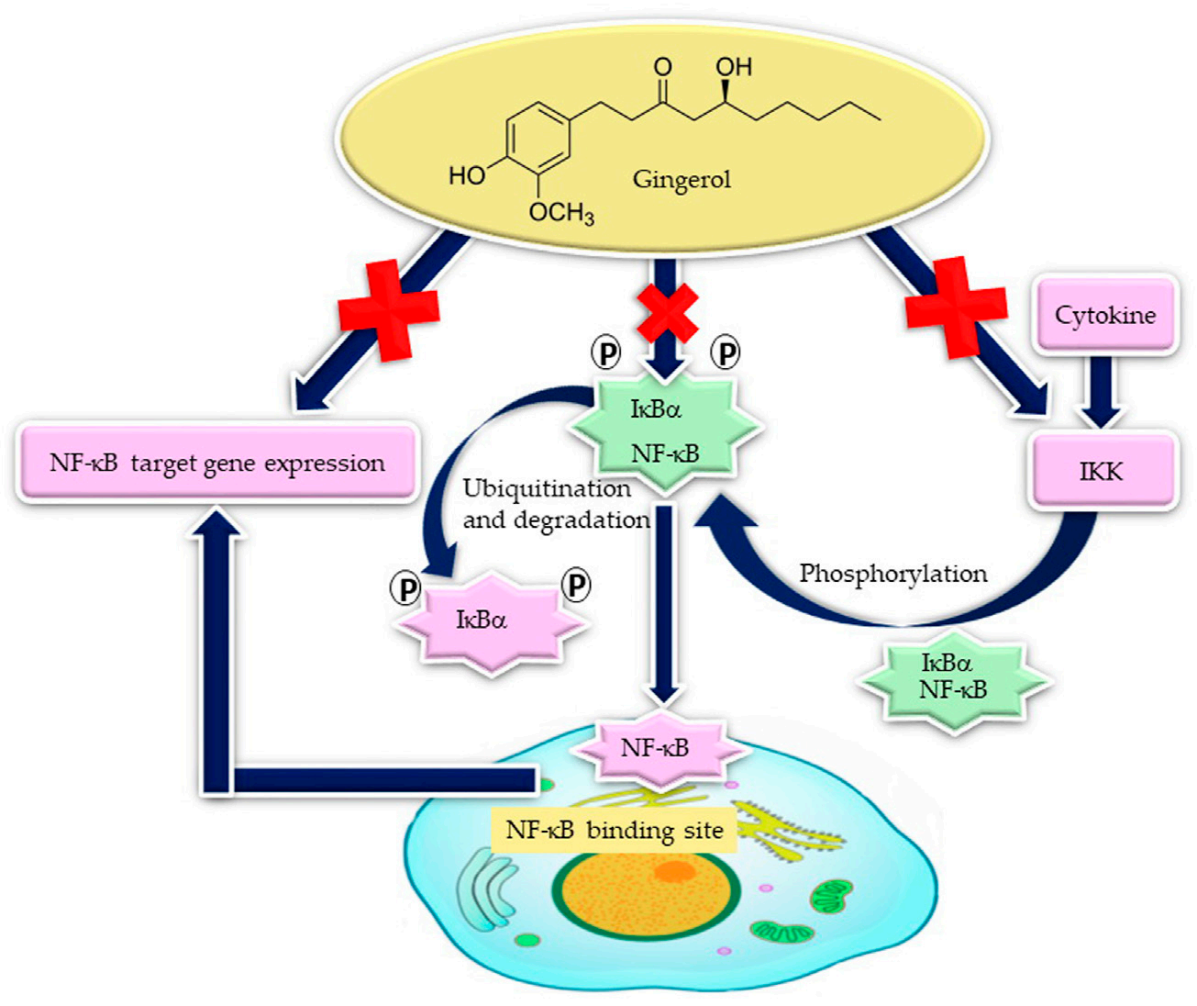
Gingerol decreases NF-кB target inflammatory gene expression by the suppression of NF-кB activity, through the stabilization of inhibitory I*к*Bα and degradation of I*к*Bα kinase (IKK) activity [adapted from the work of Li et al. (2013)].

In experimental models of non-alcoholic steatohepatitis (high-fat-diet-induced steatohepatitis), 6-gingerol has demonstrated the ability to downregulate NF-κB-mediated inflammatory responses and reduce hepatic lipogenic gene expression in hamsters (Tzeng et al., 2015). Activation of NF-κB is involved in many inflammatory ailments, including atherosclerosis, myocardial infarction, cancer, arthritis, allergy, asthma, diabetes, Crohn’s disease, Alzheimer’s disease, multiple sclerosis, osteoporosis, septic shock, psoriasis, and AIDS (Aggarwal and Shishodia, 2006). Therefore, the effectiveness of gingerols in many chronic inflammatory diseases may be associated with reduced NF-κB activity (Figure 5).

The efficacy of gingerol was measured by stimulating healthy human neutrophils with immune complexes (RNP/anti-RNP) from lupus patients or total IgG fractions from primary APS patients. Neutrophil extracellular trap (NET) release was analyzed by the enzymatic activity of NET-associated myeloperoxidase and phosphodiesterase 4 (PDE4) activity and cyclic AMP (cAMP) levels, which were also measured. In *in vivo* experiments, TLR7 agonist-induced lupus mice and aPL-expedited inferior vena cava thrombosis mice were administered 6-gingerol at a dose of 10 mg/kg/day. At low concentrations, it neutralized RNP/anti-RNP- and aPL-related NET release. Per aPL-related NET release, it was explained that 6-gingerol suppressed PDE4 activity and, thus, intracellular cAMP amounts were increased. In addition to the decrease in plasma NET levels in lupus mice treated with the TLR7 agonist, a significant decrease in anti-double-stranded DNA and anti-beta-2 glycoprotein autoantibodies was observed. In the APS model, 6-gingerol reduced plasma NET levels and neutralized the acceleration of large-vein thrombosis in human aPL (Ali et al., 2019). The same group evaluated the inhibitory effects of 6-gingerol, 8-gingerol, and 10-gingerol on NET release elicited by lupus autoantibodies. Healthy human neutrophils induced with PMA, LPS, and total IgG fractions prepared from primary APS patients (aPL) or with RNP/anti-RNP complexes from lupus patients. At doses ranging from 1 to 10 μM, both 6- and 8-gingerol exactly neutralized NETosis induced by aPL and LPS, and 10-gingerol decreased NETosis by approximately 75%. In addition, RNP/anti-RNP-induced NETosis was suppressed by 6-gingerol effectively, but all three gingerols were not found to be effective on PMA-induced NETosis (Ali and Knight, 2018).

In a skin carcinogenesis model, 6-gingerol inhibited 12-O-tetradecanoylphorbol-13-acetate (TPA)-induced epidermal ornithine decarboxylase activity and inflammation. Topical application of this compound at 2.5 µM concentration before each TPA application decreased the 7, 12-dimethylbenz[a]anthracene (DMBA)-triggered papilloma generation in mouse skin (Park et al., 1998). In 2005, in a similar study, Kim et al. (2005) have shown that the expression of COX-2 in mouse skin induced by TPA was inhibited after topical treatment of this phenolic compound. In addition, pre-application of 6-gingerol caused a reduction in both TPA-stimulated DNA binding and transcriptional activities of NF-κB through the suppression of IkappaBalpha (IκBα) disintegration and p65 nuclear translocation and avoided the TPA-stimulated phosphorylation and catalytic activity of p38 mitogen-activated protein (MAP) kinase. Previously, Yoshimi et al. (1992) also corroborated the antitumor efficacy of orally given 6-gingerol at a concentration of 0.02% for 3 weeks in the azoxymethane-induced intestinal carcinogenesis rat model.

10-Gingerol was evaluated *in vitro* in MDA-MB-231TNBC and non-tumor MCF-10A breast cancer cells and *in vivo* in the same study for its antimetastatic and antitumor effects. 10-Gingerol was capable of suppressing cell adhesion, migration, and invasion and inducing apoptosis more effectually in TNBC cells in comparison to MCF-10A cells. It has been reported that these mechanisms may also play an active role in the antitumor effect of 10-gingerol *in vivo* (Fuzer et al., 2019).

In the study of Han et al. (2022c), 6-gingerol was given orally and As_2_O_3_ intraperitoneally once a day for seven consecutive days to mice divided into five groups to investigate the potential pharmacological mechanisms of 6-gingerol on As_2_O_3_-induced myocardial injury by reducing oxidative stress, the inflammatory response, and apoptosis. Biochemical, histopathological, ELISA, and western blot analyses were performed, and As_2_O_3_ was found to induce cardiotoxicity in mice, while 6-gingerol significantly ameliorated As_2_O_3_-induced heart damage, histopathological changes, oxidative stress, myocardial mitochondrial injury, inflammation, and cardiomyocyte apoptosis, while As_2_O_3_ reversed AMP-activated protein kinase (AMPK)/silent information regulator 1(SIRT1) inhibition.

Common neurodegenerative diseases such as Alzheimer’s disease (AD) and Parkinson’s disease (PD) are characterized by neuroinflammation and protein misfolding, leading to brain damage, synaptic dysfunction, and neuronal apoptosis. In AD, there is mitochondrial dysfunction, intracellular accumulation of hyperphosphorylated tau (τ) proteins in the form of neurofibrillary tangles, and excessive accumulation of extracellular beta-amyloid plaques (Aβ), as well as oxidative stress formation due to environmental and genetic factors. It is stated that anti-inflammatory and antioxidant effects are the main feature for the possible use of ginger, especially in AD and PD (Huh et al., 2020; Arcusa et al., 2022). In animal experiments, it has been reported that ginger inhibits mRNA expression related to the expression of proinflammatory cytokines (Grzanna et al., 2004) and improves cholinergic function by decreasing oxidative stress (Sutalangka and Wattanathorn, 2017).

On the other hand, it is known that current drugs do not improve the neurodegeneration that is the basis of PD formation, but improve its symptoms, and it is said that there is no treatment that slows the progression of the disease. Oxidative stress and iron accumulation are seen with the loss of dopaminergic neurons in PD, and it is known that inflammation markers increase significantly. Oxidative stress and inflammation play an important role in the formation of both diseases. In recent years, interest in the use of these compounds has increased with the discovery of the possible beneficial effect of natural products such as ginger on the development and progression of PD. Park et al. (2013) reported that 6-shogaol protected dopaminergic cells against neurotoxicity through the inhibition of neuroinflammatory responses of microglia in *in vitro* and *in vivo* PD models. It is stated that the prominent anti-inflammatory and immunomodulatory capacity of ginger and its derivatives may be beneficial for the prevention, alleviation, and treatment of neurodegenerative diseases by improving neurological symptoms and pathological conditions by modulating cell survival signal molecules.

Although immune disorders involve more than one mechanism, the positive effects of gingerols examined in the articles summarized above have been reported in the context of numerous diseases. The effects of gingerols not only on cytokine release but also on different signaling pathways have been proven. Inhibition of activation of the NFKB pathway, which has a role in many diseases, is likely one of the main reasons for the effects of gingerols. Its apoptotic effect on cancer cells also makes this compound unique. Pharmacokinetic and clinical studies as well as formulations that will increase bioavailability are needed more of these compounds. Ginger, which has anti-inflammatory properties, and its active ingredients, such as 6-gingerol, have not been the subject of extensive clinical studies in this sense. In a randomized controlled study conducted in 2019, the effect of ginger on immunity and inflammation intermediate gene expression in patients with rheumatoid arthritis was evaluated, and it shed light on this issue. NF-κB, peroxisome proliferator-activated receptor-gamma (PPAR-γ), forkhead box P3 (FoxP3), T-box expressed in T cells (T-bet), GATA binding protein 3 (GATA-3), and retineic-receptor-related orphan nuclear receptor gamma (RORγt) gene expression were examined in patients receiving 1,500 mg of ginger powder. A significant increase in FoxP3 gene expression was observed in the ginger group. It was also shown to have a reducing effect on RORγt and T-bet gene expressions and to reduce symptoms of rheumatoid arthritis (Aryaeian et al., 2019). *In vivo* studies are summarized in Table 2.

**TABLE 2:** *In vivo* research reports of gingerols.

Compound/extract	Model	Duration of treatment	Dose	Potential mechanism	Research (author and year)
6-Gingerol	Mice infected with *Mycobacterium tuberculosis*	45 days	10 mg/kg i.p.	• Inhibited mycobacterial growth in the lung, liver, and spleen	Bhaskar et al. (2020)
				• Induced host-protective Th1 and Th17 immune responses	
6-Gingerol	LPS-induced mice	7 days	100 mg kg^−1^ body weight oral	• Decreased IL-6, TNF-α, IFN-γ, iNOS, and COX-2 mRNA expression	Guo et al. (2022)
				• Decreased caspase-3 expression	
				• Inhibited the NF-κB pathway in the ileum	
6-Gingerol	Dust mite-treated mice	7 days	10 mg/kg oral	• Decreased TNF-α, IL-6, NO, and MPO activity	Ajayi et al. (2022)
				• Reduced immune cells population	
6-Gingerol, 10-gingerol	Rat model of sepsis	Gingerols were given 2 h before, 12 h after, and 24 h after the CLP surgical operation	25 mg/kg i.p.	• Decreased IL-1β, TNF-α, and TGF-1	Rodrigues et al. (2018)
				• Reduced oxidative/nitrosative stress	
6-Gingerol, 10-gingerol enriched fraction	Aminoglycoside-induced rat nephrotoxicity model	5 days treatment	25 mg/kg, p.o., 6.25, 12.5, and 25 mg/kg i.p	• Decreased mRNA expressions of IL-2, IL-β, TNF-α, and INF-γ	Rodrigues et al. (2014)
				• Reduced lipid peroxidation and nitrosative stress	
				• Increased GS and SOD	
8-Gingerol	BALB/c mice immunized with OVA	7 days	25, 50, and 100 mg/kg i.p.	• Suppression of Con A-, LPS-, and OVA-induced splenocyte proliferation	Lu et al. (2011)
				• Decreased OVA-specific IgG, IgG1, and IgG2b levels at higher doses	
6-Gingerol	Rats immunized with sheep RBCs	7 days	800 mg/kg	• Increased lymphocyte proliferation	Farhath et al. (2013)
				• Increased humoral antibody response and cellular immunity	
6-Gingerol, 8-gingerol, 10-gingerol	Dextran sulfate sodium-induced ulcerative colitis rat model	7 days	30 mg/kg i.p.	• Decreased IL- 1β and TNF-α in serum	Zhang et al. (2017)
				• Increased SOD activity	
				• Decreased MDA levels	
				• Alleviated colitis symptoms	
6-Gingerol	Dextran sulfate sodium-induced ulcerative colitis mice model	14 days	100 mg/kg, 250 mg/kg p.o.	• Decreased mRNA levels and serum and bowel amounts of IL-10	Sheng et al. (2020)
				• Increased Th17 cells and declined Treg cells	
				• Inhibited upregulation of RORγT mRNA and protein	
6-Gingerol	Dextran sulfate sodium-induced ulcerative colitis BALB/c mice	7 days	50, 100, and 200 mg/kg p.o.	• Increased GSH, SOD, and CAT antioxidant enzymes	Ajayi et al. (2015)
				• Decreased hydrogen peroxide and MDA	
				• Decreased NO, IL-1β, and TNF-α levels	
6-Gingerol	High-fat diet-induced steatohepatitis hamster model	8 weeks	25, 50, or 100 mg/kg p.o.	• Decreased levels of MCP-1, TNF-α, IL-1β, and IL-6	Tzeng et al. (2015)
				• Reduced nuclear NF-κB p65 protein	
6-Gingerol	Mice with tumor promoter-induced inflammation	30 min prior to the application of TPA	10 µmol	• Suppressed TPA-induced epidermal ornithine decarboxylase activity and inflammation	Park et al. (1998)
6-Gingerol	TPA-induced mice	30 min prior to the application of TPA	5 or 30 μmol	• Reduction in transcriptional activities of NF-κB	Kim et al. (2005)
				• Avoided catalytic activity of p38 mitogen-activated kinase	

## Using nanoformulations for improvement of therapeutic effects and bioavailability of gingerols

The bioavailability of gingerols, especially the efficacy of 6-gingerol in drug therapy, is limited owing to their poor water solubility (Wang et al., 2018a). Possible technological approaches to increase the solubility and bioavailability of gingerols and to prevent harmful interactions include micro/nanocarriers, such as proliposomes, exosome-like nanoparticles, nanoparticles, and micro/nanoemulsions, which have the potential to increase their efficacy and stability, to reduce their toxicity, and to maintain sustained release. The National Cancer Institute (NCI) has accepted nanotechnology for treatment as an emerging field with the possibility to revolutionize modern medicine (Nair et al., 2010; Taghipour et al., 2018).

Several studies have addressed the anti-inflammatory effect of gingerols by nanoformulation, but their numbers are limited. Baskar et al. (2012) developed ultra-deformable nanovesicles for transdermal administration. A permeation study was performed using the *in vitro* dermal membrane system relating the ear skin of *Capra aegagrus hircus*, and the absorbance value of the collected samples was measured using a UV-Vis spectrophotometer at 282 nm. The findings showed that drug penetration alone was lower compared to vesicular-mediated drug release. The ultra-flexible or ultra-deformable vesicle system was more effective in the deliver of gingerol to the depths of the skin with an 85% encapsulation efficiency of gingerol.

Liposomes show stability problems, such as fusion, aggregation, precipitation, phospholipid oxidation and hydrolysis, drug leakage, and formulation difficulties arising during transportation and storage processes. Proliposomes were discovered in 1986 and are a dry and free-flowing granular powder preparation based on drugs, phospholipids, and other additives, formulated to form a liposomal suspension, to overcome the disadvantages of liposomes in stability. Proliposomes can be formulated directly into oral dosage forms that are easily prepared at an industrial scale. Higher physical and chemical stability is achieved with proliposomes compared to liposomes. Wang et al. (2009) addressed the solubility and oral bioavailability of 6-gingerol in proliposomes. With 6-gingerol proliposomes, the physicochemical stability and *in vitro* release rate were considerably increased compared to the free drug, and oral bioavailability increased 5-fold *in vivo* (Wang et al., 2018c). The size and zeta potential (ZP) of the nanocarriers determine the absorption into various tissues and organs in the body. Proliposomes with spherical morphology and no particle aggregation were characterized morphologically with transmission electron microscopy (TEM), and their ZP was −3.61 mV, indicating a relatively homogeneous distribution. The physical stability of liposomes is affected by storage conditions, and aggregation is noted at high temperatures. Particle size is an important parameter with respect to stability. In addition, negative ZP improves the electrostatic stabilization of the dispersion, preventing vesicle fusion and aggregation (Yücel et al., 2018). It was noted that nanoformulations <100 nm in diameter could escape macrophage clearance. In this study, 6-gingerol proliposomes reduced the clearance by macrophages, contributing to increased circulation in blood plasma. The pharmacokinetic results showed that the 6-gingerol encapsulated in proliposomes was retained in the bloodstream for much longer than the free drug.

The low solubility of 6-gingerol is caused by its poor oral absorption and fast metabolism, and this situation limits biological applications such as anti-inflammatory effect. In this study, the alternative formulations for oral administration called the self-microemulsifying drug delivery system (SMEDDS), formed by oil, surfactant, and co-surfactant or water, were prepared with stable physicochemical belongings such as the mean droplet size, ZP, and encapsulation efficiency. The *in vitro* release of 6-gingerol from the delivery system in the three different media was significantly higher than in the free 6-gingerol. A pharmacokinetic study in rats showed that the SMEDDS effectively increased and prolonged plasma concentration, with a 6.58-fold increase in oral bioavailability compared with the free 6-gingerol (Xu et al., 2016). For the evaluation of SMEDDS, droplet size and polarity are critical parameters as they determine the overall emulsification rate and scope of absorption, as well as drug release. Smaller droplet size provides effective drug absorption and improved oral bioavailability. In addition, the low polydispersity index (PDI) also shows the uniformity of particle size in nanocarriers. It was reported that 6-gingerol-SMEDDS, which have a narrow size distribution and are electrically and physically stable, have greater surface area due to their small particle size and that 6-gingerol was efficiently released from SMEDDS for a greater duration. In the pharmacokinetic studies, the SMEDDS accelerated the intestinal lymphatic transport of 6-gingerol protected from enzymatic degradation and inhibitory dispersion of the component, increasing oral absorption. The *in vivo* absorption of 6-gingerol can be predicted by observing the *in vitro* release profiles. The lack of a comparable *in vitro–in vivo* correlation study made this study innovative. The objective of *in vitro–in vivo* correlation is to serve as a surrogate for the *in vivo* bioavailability study and to support and/or validate the use of dissolution methods and specification settings and to assist in quality control during scale-up and post-approval modifications. Level A, *in vitro–in vivo* correlation was achieved per Food Drug Administration (FDA) guidelines. Level A which is linear most commonly used as point-to-point correlation is developed and represents the rate of absorption of the drug *in vitro* and *in vivo* (Narayanasamy and Shabaraya, 2017).

Wei et al. (2018) developed an optimized nanostructure lipid carrier (NLCs) of 6-gingerol to improve its poor water solubility and limited oral bioavailability. Solid lipid (glyceryl monostearate), another liquid lipid (decanoyl/octanoyl-glycerides), and mixed surfactants (Poloxamer 188 and Tween 80) were used to prepare NLCs by the high-pressure homogenization method. The characterization of formulation was assessed for mean particle size, ZP, encapsulation efficiency, and *in vitro* drug release. Pharmacokinetic parameters such as the area under the concentration–time curve (AUC) and mean residence time (MRT) were also evaluated in rats. The optimal NLCs containing 6-gingerol formulation displayed a homogenous spherical shape with a mean particle size and ZP of 63.59 ± 5.54 nm and −12.18 ± 1.06 mV, respectively. Encapsulation efficacy and drug loading were 76.71 ± 1.11 and 1.17 ± 0.35%, respectively. The *in vitro* release profile of 6-gingerol was sustained. Small particle size and low PDI may affect the uptake of the reticuloendothelial system and, thus, oral absorption and bioavailability, leading to passive accumulation in tissues. Given its hydrophobic character of 6-gingerol high solubility in lipids and its dispersion in the particle matrix, high loading efficiency was achieved. The authors implied that the sustained *in vitro* release feature of the formulation can provide an effective and *in vivo* treatment and increase its bioavailability. Indeed, drug concentrations in serum, MRT, and AUC0-t were significantly increased after oral administration, related to the free drug. These results showed that NLC formulation containing 6-gingerol could be effective in prolonging the *in vivo* effect time of the drug with sustained drug release and oral bioavailability.

Several studies addressed the anti-inflammatory activity and different nanoformulations. The pharmacological treatment of osteoarthritis with non-steroidal anti-inflammatory agents includes acetaminophen, aspirin, diclofenac, and selective COX-2 inhibitors. Given their several limitations, such as gastrointestinal side effects and adverse cardiac events, herbal supplements, such as ginger, are needed to provide alternative therapies. Ginger’s anti-inflammatory properties are caused by the inhibition of several gene-encoding cytokines, chemokines, COX-1, and COX-2, thus modulating the biochemical pathways activated in chronic inflammatory processes. Amorndoljai et al. (2017) prepared NLCs in various compositions of solid lipid, liquid lipid, surfactant, and water mix with the ginger extract at 5%. This technique provided increased biodegradability and biocompatibility and was non-toxic. Developed NLCs were compared with 1% diclofenac gel for 12 weeks in patients with knee osteoarthritis for topical relief of pain. One hundred and eighteen people participated, and both ginger extract in NLCs and diclofenac gel improved stiffness, knee pain, and physical function following 12 weeks of treatment. Higher response rate for at least 50% reduction in pain was found following ginger extract in NLC compared to topical diclofenac. In an earlier study, plysersic gel (combination of ginger and galangal) showed that a combination of ginger and plai led to stronger anti-inflammatory activity.

In another study, microemulsion and microemulsion gel formulations were prepared with ginger extract containing 6-gingerol for appraisal of the anti-inflammatory activity of ginger extract. Physicochemical characterization, stability, and release studies of microemulsion formulations were also performed. The anti-inflammatory activity of ginger extracts was investigated with a carrageenan-induced rat paw edema test and compared with the efficacy of indomethacin. The best region to create microemulsion with a pseudo-ternary phase diagram was determined by the water titration method. It is known that all natural gum derivatives are used for the preparation of microemulsion gels, whereas certain cellulose derivatives (carboxymethylcellulose sodium) and polyacrylic derivatives (Carbopol 934 and Carbopol 940) are used. The findings showed that the inclusion of 5% w/w ginger extract in microemulsions and microemulsion gels caused a slight decrease in pH and viscosity change, but the ginger extract showed reasonable stability in terms of phase separation or precipitation. Six heating–cooling cycles were applied, and next, the content of 6-gingerol in the microemulsion and microemulsion gel decreased to 85.19%–92.21% and 92.87%–97.28%, respectively. The release of 6-gingerol from microemulsions and microemulsion gels was evaluated by comparison with the solution. The cumulative amount of released 6-gingerol from microemulsions and microemulsion gels at 24 h was 33.52%–45.76% and 27.58%–37.33%, respectively. All release profiles showed the best agreement with the diffusion-controlled Higuchi kinetics. Due to the higher viscosity of the microemulsion gel, the release was delayed and the release rate was lower (*p* < 0.05). Additionally, the formulation ingredients also had an effect on the release rate. The microemulsion gel formulation using Cremophor^®^ RH 40 and Carbopol 940 containing 5% w/w ginger extract showed a slower onset of anti-inflammatory activity and a longer duration of anti-inflammatory activity compared to the microemulsion (Praditbongkotch, 2009).

Another microemulsion formulation was prepared by Akram et al. (2019) where the ginger extract was evaluated for its efficacy in *in vitro* anti-inflammatory activity. Enhancement of the solubility and stability of ginger extract with microemulsion formulation and evaluation of its anti-inflammatory activity were the major objectives of this study. For the optimal components for microemulsion, isopropyl myristate, PEG 400, and Tween 80 were selected as the oil phase, surfactant, and co-surfactant, respectively. The pH, viscosity, refractive index, conductivity, droplet size, ZP, PDI, and ginger extract content of microemulsions were evaluated. The optimal formulation containing 4% isopropyl myristate, 40% of surfactant/co-surfactant ratio, and 56% water displayed the best chemical and physical properties with small globular size and low viscosity. It also showed an anti-inflammatory effect (higher inhibition of protein denaturation) significantly (*p* < 0.05) as compared to the reference drug, piroxicam solution. The authors concluded that a stable microemulsion system could be developed with ginger extract and it showed promise in anti-inflammatory activity. Microemulsions are transparent, isotropic, thermodynamically stable systems and have micro-sized spherical droplets with oil, water, and surfactant and usually a co-surfactant, with very low interface tension between oil and water phases. Tissue barrier must be overcome in order to achieve therapeutic effects of drugs by topical and transdermal routes. Microemulsions (MEs) are used as topical and transdermal drug delivery systems to achieve local effect and to increase drug penetration through the skin. Low viscosity of MEs can cause skin applicability problem. For this purpose, ME gel formulations were also prepared in order to increase the viscosity and the residence time on the skin. Determining the optimum formulation components to reach the correct formulation will directly affect the stability and effectiveness of the formulation (Praditbongkotch, 2009; Akram et al., 2019).

The two studies mentioned above, microemulsion and microemulsion gel compared to the unformulated drug, improved the stability and solubility of the drug, leading to increased efficacy. As a novel approach to solubility, bioavailability, and stability problems, nanoparticle-based phytosome complexed with chitosan for gingerol delivery was developed by Singh et al. (2018). The phytosome was prepared by mixing gingerol with soya lecithin and loaded in the aqueous solution of chitosan to formulate the phytosome complexed with chitosan. Formulations were characterized for their entrapment efficiency, drug loading and particle size, and physical compatibility, corroborating the inhibition of particle aggregation with the chitosan complex. Scanning electron microscopy (SEM) analysis was used for the morphologies of optimized phytosome complexed with chitosan and phytosomes. An *in vitro* anti-inflammatory study was carried out with the human red blood cell membrane stabilization and albumin denaturation method. Gingerol, phytosome complexed with chitosan, phytosomes, the mixture of gingerol and chitosan, chitosan alone, and aspirin as a reference positive control were used for the *ex vivo* anti-inflammatory activity. Phytosome complexed with chitosan showed effective anti-inflammatory activity (a series of actions that prevent shrinkage of cells as well as heat-induced protein denaturation that activates or enhances the release of the intracellular part) compared to phytosome against inflammation that causes respiratory infections. Phytosome is one of the lipid-based vesicular delivery systems that can be used for encapsulation of drugs and plant-derived nutraceuticals and reducing the problems with solubility and bioavailability. The phytosome structure developed by the complex formation of phospholipid and plant-derived compounds or extracts has been used with many compounds, such as *Ginkgo biloba* L., grape seed, hawthorn, milk thistle, green tea, and ginseng (Ajazuddin, 2010), and stands out among the best strategies to increase bioavailability. Chitosan was chosen as a biodegradable, biocompatible, and positively charged hydrophilic polymer in order to increase the absorption rate of gingerol (by opening tight junctions between epithelial cells). Soy lecithin is a suitable phospholipid for chitosan due to its negatively charged ions to make and develop the cell-like complex structure of gingerol. The spherical and oval structure containing gingerol, soy lecithin, and chitosan form a complex structure. Chitosan concentration had a significant effect on the size of the phytosome complex of gingerol. Also, from the pharmacokinetic study, the phytosome–chitosan complex containing gingerol showed maximum plasma concentration (C_max_), the time to maximum plasma concentration (T_max_), and AUC0-∞, as well as half-life (t_1/2_) (pharmacokinetic parameters), compared to non-complexed phytosome. These results showed that the phytosome–chitosan complex has a better sustained release profile and increased oral absorption of gingerol. Thus, the phytosome–chitosan complex delivery system is suitable for oral administration of gingerol. Furthermore, studies with the phytosome–chitosan complex of ginger established that optimized formulations prevent cells from shrinking, inhibit a range of actions, and prevent heat-induced protein denaturation. The phytosome–chitosan complex approach was also associated with greater efficacy of ginger, increasing protein binding, acting as free radical scavenger, and decreasing the immune function with the free radical formation in the treatment of respiratory infections (Singh et al., 2018).

Exosomes are small vesicles that can transport mRNA, microRNA (miRNA), bioactive lipids, and proteins, are produced by various mammalian cells (such as immune, epithelial, and tumor cells), and are involved in intercellular communication. Exosome-like nanoparticles are taken up by intestinal macrophages and stem cells. Mu et al. (2014) evaluated the biological effects of exosome-like nanoparticles obtained from edible plants on mammalian cells. Exosome-like nanoparticles were isolated from ginger, carrot, and grapefruit, and the results showed that they have a similar size and structure to mammalian-derived exosomes. Studies with exosome-like nanoparticles isolated from ginger showed that they specifically induced nuclear translocation of macrophage Nrf2, which is a key regulator of the heme oxygenase (HO-1) gene and has an important role in anti-inflammation and antioxidation and intestinal wingless/integrated (Wnt)/T-cell factor 4 (TCF4) activation (Ajayi et al., 2015). Exosomes are nanoparticles released in various diseased as well healthy cells, obtained from body fluids by various centrifugation techniques. The stage of the disease can be determined by the changes in the amount and structure of the exosomes obtained from the patient and has potential as drug delivery system for the future. In this study, it has been noted that ginger-derived exosome-like nanoparticles played a significant role in regulating the levels of anti-inflammation cytokines, antioxidant effect inducing the expression of genes, and maintaining intestinal homeostasis. Compared with exosomes from other plants, ginger-derived exosome-like nanoparticles induced Nrf2 nuclear translocation more potently in mouse macrophage (Raw 264.7) cells.

Exosome-like nanoparticles have shown improved therapeutic effect. This approach is advantageous as an alternative to traditional clinical application. Zhang et al. (2016) by characterizing the structures of isolated ginger-derived nanoparticles, explored the possibility of their therapeutic efficacy in inflammatory bowel disease and colitis-associated cancer. The authors characterized nanoparticles with an average size of ∼230 nm and negative ZP derived from edible ginger and revealed effective colonic targeting of nanoparticles containing high levels of lipids, several proteins, ∼125 miRNAs, and large amounts of ginger bioactive components (6-gingerol and 6-shogaol) following oral administration. Increased uptake of nanoparticles derived from edible ginger by intestinal epithelial cells and macrophages was noted, and the formulation was non-toxic. In other mouse colitis models, nanoparticles decreased the expression of proinflammatory cytokines (IL-6, IL-1β, and TNF-α) and acute colitis, elevated the expression of anti-inflammatory cytokines (IL-10 and IL-22), and enhanced intestinal repair, establishing efficacy in preventing chronic colitis and colitis-related cancer. The production of medical nanoparticles with plants is a new approach to the treatment of inflammatory bowel disease. The use of ginger-derived nanoparticles orally affords an alternative to most drugs associated with serious side effects that can be administered only orally. Targeting of ginger-derived nanoparticles to the colon, the site of intestinal inflammation in ulcerative colitis, was not associated with local or systemic side effects. Findings in this study are in agreement with others showing that nanoparticles administered orally are stable during transit through the stomach and target the small intestine and colon. Moreover, isolated and identified nanoparticles have monodispersed size distribution which is a critical parameter for the effective design of nanoparticles. Characterization of ginger-derived nanoparticles established a similar range to other isolated edible nanoparticles from grapes and grapefruit. These systems, exemplified by ginger-derived nanoparticles, are adaptable to a large-scale production and provide an effective therapeutic strategy for the prevention and treatment of inflammatory bowel disease. The studies carried out are summarized in Table 3.

**TABLE 3:** Nanoformulations with gingerol/ginger extract.

Nanoformulation	Compound/extract	Experimental model	Results	Research (author and year)
Nanovesicles	Gingerol	*In vitro* dermal membrane system	• Improved drug penetration	Baskar et al. (2012)
			• Enhanced gingerol delivery to the depths of the skin	
Proliposomes	6-Gingerol	*In vitro* drug *release*	• Higher physical and chemical stability	Wang et al. (2018c)
		*In vivo* tumor model (rat)	• Increased oral bioavailability	
			• Improved antitumor effect	
Self-microemulsifying drug delivery system		*In vitro* drug release, *in vivo* (rat)	• Improved the prolonged plasma concentration	Xu et al. (2016)
			• Increase in oral bioavailability	
Nanostructure lipid carrier		*In vivo* (rat)	• Increased concentrations in serum, MRT, and AUC0-t	Wei et al. (2018)
			• Enhanced oral bioavailability	
Nanostructure lipid carrier	Ginger extract	*In vivo*, clinical trial (patients with knee osteoarthritis)	• Increased biodegradability and biocompatibility	Amorndoljai et al. (2017)
			• At least 50% reduction in pain with NLC treatment compared to topical diclofenac	
Microemulsion and microemulsion gel formulations		*In vivo* (rat)	• Microemulsion gel formulation showed a slower onset of anti-inflammatory activity and a longer duration	Praditbongkotch, (2009)
Microemulsion		*In vitro*	• Enhanced solubility and stability	Akram et al. (2019)
			• Higher inhibition of protein denaturation	
Phytosome complexed with chitosan	Gingerol		• Effective anti-inflammatory activity compared to phytosome	Zhang et al. (2017)
			• Maximum C_max_, T_max_, AUC0-∞ as well as T_1/2_ (pharmacokinetic parameters) compared to non-complex phytosome	
			• Sustained release profile and increased oral absorption of gingerol	
Exosome-like nanoparticles	Ginger		• Induced Nrf2	Mu et al. (2014)
			• Activation of intestinal Wnt/TCF4	
Ginger-derived nanoparticles		*In vivo* (mouse)	• Decreased expressions of IL-6, IL-1β, and TNF-α	Zhang et al. (2016)
			• Enhanced intestinal repair	
			• Effective in preventing chronic colitis and colitis-related cancer	

## Conclusion

The main purpose of studies in the field of medicine has been to develop a novel molecule to treat diseases. To meet this goal, focus has been on improving bioavailability and solubility, reducing the drug dose, extending the dosing interval, reducing side effects, and ensuring the drug reaches its target area whatever the delivery systems are (Nair et al., 2010; Patra et al., 2018). Traditional application of natural compounds has routinely failed given issues with solubility, permeability, and bioavailability. To overcome these problems, novel drug delivery systems and therapeutic approaches have been developed with a major focus on natural compounds and nanotechnology as a viable strategy to advance the physicochemical properties of these compounds (Nair et al., 2010).

Gingerols are the most abundant active ingredients of ginger, with 31 gingerol-related compounds identified so far. Among them, 6-gingerol is the main pungent and bioactive component. Apart from 4-, 6-, 8-, and 10-gingerols, other bioactive components contained in the rhizome also have anti-inflammatory activity. These compounds are 10-gingerol, 1-dehydro-10-gingerdione, 10-gingerdione, gingerfilenone A hexahydrocurcumin, and tetrahydrocurcumin (Braga, 2019). Previous studies focused on developed nanocarriers with 6-gingerol. Generally, they aimed to develop nanoformulations that show improved bioavailability and increased efficacy, eliminating physicochemical stability problems.

The immunomodulatory effects of gingerols and shogaols and their nanodrug delivery systems discussed herein corroborate the nutraceutical value of ginger rhizome and its application as a natural origin drug. Its administration in humans offers an alternative to synthetic drugs, which often have serious side effects. While a wide range of pharmacological studies focused on ginger show that the spice has functional food value and may offer therapeutic efficacy, further studies could be profitable directed at broadening the understanding of its mechanisms within the context of numerous diseases.
